# Key Disease Mechanisms Linked to Amyotrophic Lateral Sclerosis in Spinal Cord Motor Neurons

**DOI:** 10.3389/fnmol.2022.825031

**Published:** 2022-03-15

**Authors:** Virginie Bottero, Jose A. Santiago, James P. Quinn, Judith A. Potashkin

**Affiliations:** ^1^Chicago Medical School, Rosalind Franklin University of Medicine and Science, Center for Neurodegenerative Diseases and Therapeutics, Discipline of Cellular and Molecular Pharmacology, North Chicago, IL, United States; ^2^NeuroHub Analytics, LLC, Chicago, IL, United States; ^3^Q Regulating Systems, LLC, Gurnee, IL, United States

**Keywords:** ALS, amyotrophic lateral sclerosis, co-expression networks, network analysis, neurodegeneration, switch genes, motor neuron disease

## Abstract

Amyotrophic lateral sclerosis (ALS) is a fatal neurodegenerative disease with no modifying treatments available. The molecular mechanisms underpinning disease pathogenesis are not fully understood. Recent studies have employed co-expression networks to identify key genes, known as “switch genes”, responsible for dramatic transcriptional changes in the blood of ALS patients. In this study, we directly investigate the root cause of ALS by examining the changes in gene expression in motor neurons that degenerate in patients. Co-expression networks identified in ALS patients’ spinal cord motor neurons revealed 610 switch genes in seven independent microarrays. Switch genes were enriched in several pathways, including viral carcinogenesis, PI3K-Akt, focal adhesion, proteoglycans in cancer, colorectal cancer, and thyroid hormone signaling. Transcription factors ELK1 and GATA2 were identified as key master regulators of the switch genes. Protein-chemical network analysis identified valproic acid, cyclosporine, estradiol, acetaminophen, quercetin, and carbamazepine as potential therapeutics for ALS. Furthermore, the chemical analysis identified metals and organic compounds including, arsenic, copper, nickel, and benzo(a)pyrene as possible mediators of neurodegeneration. The identification of switch genes provides insights into previously unknown biological pathways associated with ALS.

## Introduction

Amyotrophic lateral sclerosis, also known as motor neuron disease, is a fatal neurodegenerative disease affecting the upper and lower motor neurons leading to muscle atrophy, paralysis, and death, usually within 2–5 years from symptoms onset (Ingre et al., 2015). Currently, there are no disease-modifying therapies available. Riluzole and edaravone are the only drugs approved for disease management; however, these drugs produce only modest effects on symptoms and life expectancy (Nagoshi et al., 2015). The lack of sensitive and specific biomarkers has been a significant hurdle in the development of therapeutic strategies. To date, the most promising biomarkers are the neurofilament light (NfL) and the phosphorylated neurofilament heavy subunit (pNFh). Several studies have reported higher expression levels of NfL and pNFh in the cerebrospinal fluid (CSF) and blood of ALS patients compared to healthy controls (Boylan et al., 2013; Benatar et al., 2018). Nonetheless, these markers have been reported as helpful in identifying patients with Alzheimer’s disease, thereby limiting the specificity of diagnosing ALS (Preische et al., 2019).

Epidemiological studies have indicated numerous risk factors associated with ALS, including older age, male sex, family history, diabetes, dietary factors, and physical fitness, suggesting the disease is mainly multifactorial (McCombe and Henderson, 2010; Ingre et al., 2015; Couratier et al., 2016). Indeed, around 90%–95% of the cases are sporadic. The most common mutations in ALS are the hexanucleotide repeat expansion in the C9ORF72 gene and polymorphisms in superoxide dismutase (SOD1; Hardiman et al., 2017). In addition, genome-wide association studies have identified more than 30 genetic factors implicated in ALS, reflecting the complexity and multifactorial nature of the disease (Hardiman et al., 2017).

Several molecular mechanisms have been implicated in the pathogenesis and progression of ALS. Inflammation, mitochondrial dysfunction, protein misfolding, RNA metabolism, and lipid metabolism are examples of a wide range of dysregulated pathways identified in ALS studies. Network-based analysis has been instrumental in elucidating disturbances in these pathways. For instance, a transcriptomic meta-analysis identified the dysregulation of lipid metabolism as an early pathological alteration in the spinal cord of SOD1 mice (Fernandez-Beltran et al., 2021). Interestingly, lipid metabolic pathways, including cholesterol biosynthesis, ceramide metabolism, and eicosanoid synthesis, were associated with disease progression. Furthermore, interactome and transcription factor analysis from human pluripotent stem cells from ALS patients with FUS and SOD1 mutations revealed unique pathways associated with these genetic mutations. For example, herpes simplex virus infection was predominantly associated with FUS mutations, whereas dysregulation of metabolic pathways and neuroactive ligand-receptor interactions were connected to mutations in SOD1 (Dash et al., 2020). In addition, an integrative transcriptomic analysis identified 15 small bioactive molecules as potential drug candidates for ALS (Park et al., 2021).

Recently, analysis of co-expression networks to identify genes associated with drastic gene expression changes, best known as “switch genes”, has shown promise in identifying disease mechanisms in several neurodegenerative diseases. For example, co-expression network analyses identified key switch genes in the brain of Alzheimer’s disease, frontotemporal dementia, and vascular dementia (Potashkin et al., 2019, 2020). More relevant to the present study, analysis of co-expression networks identified sex-specific switch genes and pathways in the blood of ALS patients (Santiago et al., 2021). This study expands our analyses to identify switch genes and pathways in spinal cord motor neurons in ALS patients. The identification of switch genes may unveil previously unknown mechanisms in the development of ALS.

## Materials and Methods

### Database Mining

The NCBI GEO database^1^ and ArrayExpress database^2^ were searched on August 2021 for studies in which transcriptomic data were available from ALS patients. The database was queried using the search terms “ALS”, “neuron”, and “Homo sapiens” (Organism) for the study types expression profiling by array and expression profiling by high-throughput sequencing. Only datasets containing transcriptomic data from spinal cord motor neurons were selected for analysis. Seven arrays containing samples from patients’ spinal cords were identified (Table 1). GSE52946 was the only high throughput sequencing study included in this study. The sample population of each dataset is presented in Table 2. Raw data from the expression arrays were imported into SWIM. The SWIM algorithm consists of several steps, as we previously described (Potashkin et al., 2019, 2020). The fold-change thresholds used were 2, 3, 4, 1.5, 2.5, 1.75, and 4.5 for the arrays GSE833, GSE19332, GSE20589, GSE26927, GSE56500, GSE68605, and GSE52946, respectively. Different thresholds were used to obtain a significant number of switch genes from each array.

**Table 1:** Gene expression studies analyzed by the switch miner software.

Arrays	Platform	Description	ALS/control	References
GSE833	A-AFFY-32 - Affymetrix GeneChip HuGeneFL Array	Gray matter of lumbar spinal cord. Familial and sporadic.	6/4	Dangond et al. (2004)
GSE19332	A-AFFY-44 - Affymetrix GeneChip Human Genome U133 Plus 2.0	Isolated motor neurons in CHMP2B-related ALS cases	3/7	Cox et al. (2010)
GSE20589	A-AFFY-44 - Affymetrix GeneChip Human Genome U133 Plus 2.0	Motor neuron from cervical spinal cord in SOD1-ALS.	3/7	Kirby et al. (2011)
GSE26927	A-MEXP-931 - Illumina HumanRef-8 v2 Expression BeadChip	Cervical spinal cord	10/10	Durrenberger et al. (2012, 2015)
GSE56500	A-AFFY-143 - Affymetrix GeneChip Human Exon 1.0 ST Array version 1	Lower motor neurons laser-microdissected from spinal cords of sporadic or familial ALS patients	6/6	Highley et al. (2014)
GSE68605	A-AFFY-44 - Affymetrix GeneChip Human Genome U133 Plus 2.0	laser captured lower motor neurons from ALS with C9ORF72 mutation.	8/3	Cooper-Knock et al. (2015)
GSE52946	GPL11154 Illumina HiSeq2000 (Homo Sapiens)	Whole lumbar spinal cord homogenate	10/10	Butovsky et al. (2015)

**Table 2:** Sample population.

Arrays	Samples	# samples	Age (years)	PMI (h)	Disease duration (years)	Gender (F/M)
GSE833	Control	4	56	10	-	NA
	ALS	6	61	10	NA	NA
GSE19332	Control	7	NA	NA	-	NA
	ALS	3	NA	NA	NA	NA
GSE20589	Control	7	69	17	-	3/4
	ALS	3	55	33	1.9	3/0
GSE26927	Control	10	67	9	-	0/10
	ALS	10	68	28	2.45^*^	3/7
GSE56500	Control	6	62	NA	-	1/5
	ALS	6	60	NA	NA	2/4
GSE68605	Control	8	60	NA	-	3/1
	ALS	3	66	NA	2.1	5/3
GSE52946	Control	10	53	NA	-	NA
	sALS	10	58	NA	1.9	2/8
	fALS	4	37	NA	0.75	1/3

PMI: Postmortem interval in hours. ^*^The disease duration was disclosed for 6 out of 10 patients only. NA: not available.

### Swim Analysis to Identify Switch Genes

Raw gene expression data from the seven arrays were imported into SWIM. The SWIM algorithm consists of several steps. Briefly, in the preprocessing stage, genes with no or low expression are removed. In the filtering step, the fold changes were set for each array, and genes that were not significantly expressed between ALS subjects compared to controls are removed. The False discovery rate method (FDR) was used for multiple test corrections. Pearson correlation analysis was performed to build a co-expression network of genes differentially expressed between ALS subjects and controls. The k-means algorithm was used to identify communities within the network as previously demonstrated (Fiscon et al., 2018). SWIM uses a Scree plot to determine the number of clusters and the clusters with the lowest number of sum of the square error (SSE) values among the replicates is designated as the number of clusters. A heat cartography map is built using a clusterphobic coefficient *Kπ* and the global-within module degree Zg. The coefficient *Kπ* measures the external and internal node connections whereas *Zg* measures the extent each node is connected to others in its community. A node is considered a hub when Zg > 5. The average Pearson correlation coefficient (APCC) between the expression profile of each node and its nearest neighbors is used to build the heat cartography map. Using the APCC, three types of hubs are defined; date hubs that show low positive co-expression with their partners (low APCC), party hubs that show high positive co-expression (high APCC), and nodes that have negative APCC values are called fight-club hubs. In the final step, switch genes are identified that are a subset of the fight-club hubs that interact outside of their community. Switch genes are defined as not being a hub in their cluster (low *Zg* <2.5), having many links outside their cluster (*Kπ* >0.8, when *Kπ* is close to 1 most of its links are external to its module), and having a negative average weight of incident links (APCC <0). Switch genes are defined as the set of genes that interact outside their community, are not in local hubs and, are mainly anti-correlated with their interaction partners (Fiscon et al., 2018).

### Pathway Enrichment Analysis

Official gene symbols for the switch genes were imported into NetworkAnalyst for network and pathway analyses (Zhou et al., 2019). Each dataset of switch genes was analyzed separately. The minimum connected network was used for analysis. The Kyoto Encyclopedia of Genes and Genome (KEGG) pathway database was used as an annotation source (Kanehisa and Goto, 2000).

### Gene-Transcription Factors Interaction Analysis

Gene-transcription factors interactome was performed in NetworkAnalyst. Transcription factor and gene target data were derived from the Encyclopedia of DNA Elements (ENCODE) ChIP-seq data, ChIP Enrichment Analysis (ChEA), or JASPAR database (Lachmann et al., 2010; Wang et al., 2013; Fornes et al., 2020). ENCODE uses the BETA Minus Algorithm in which only peak intensity signal <500 and the predicted regulatory potential score <1 is used. ChEA transcription factor targets database inferred from integrating literature curated Chip-X data. JASPAR is an open-access database of curated, non-redundant transcription factor-binding profiles. A Venn diagram analysis was performed with the transcription factors identified with each database. Transcription factors were ranked according to network topology measurements, including degree and betweenness centrality.

### Gene-miRNA Interaction Analysis

The gene-miRNA interactome was performed in NetworkAnalyst. The Gene-miRNA interactome was carried out from comprehensive experimentally validated miRNA-gene interaction data collected from TarBase v.8.0 and miRTarBase v.8.0 (Chou et al., 2016, 2018; Karagkouni et al., 2018). miRNAs were ranked according to network topology measurements such as degree and betweenness centrality. Venn diagram analysis was used to identify the shared and unique set of miRNAs between ALS analyses. miTALOS 2.0 localized miRNA targets in signaling pathways (Kowarsch et al., 2011; Preusse et al., 2016). This software is publicly available and can be accessed at http://mips.helmholtz-muenchen.de/mitalos/#/search.

The functional analysis of the miRNA was performed using the prediction tool TargetScan from the miTALOS 2.0 website accessible at http://mips.helmholtz-muenchen.de/mitalos/#/search (Kowarsch et al., 2011; Preusse et al., 2016).

### Gene-Chemical Analysis

Protein-chemical associated analysis was performed in NetworkAnalyst, which uses the literature curated gene-chemical database Comparative Toxicogenomics, a genomic resource available to the public that is derived from genes and proteins of toxicologic significance to humans (Mattingly et al., 2003). Chemicals were ranked according to network topology measurements, degree, and betweenness centrality.

## Results

### Database Mining for ALS Transcriptomic Studies

**Figure 1:**
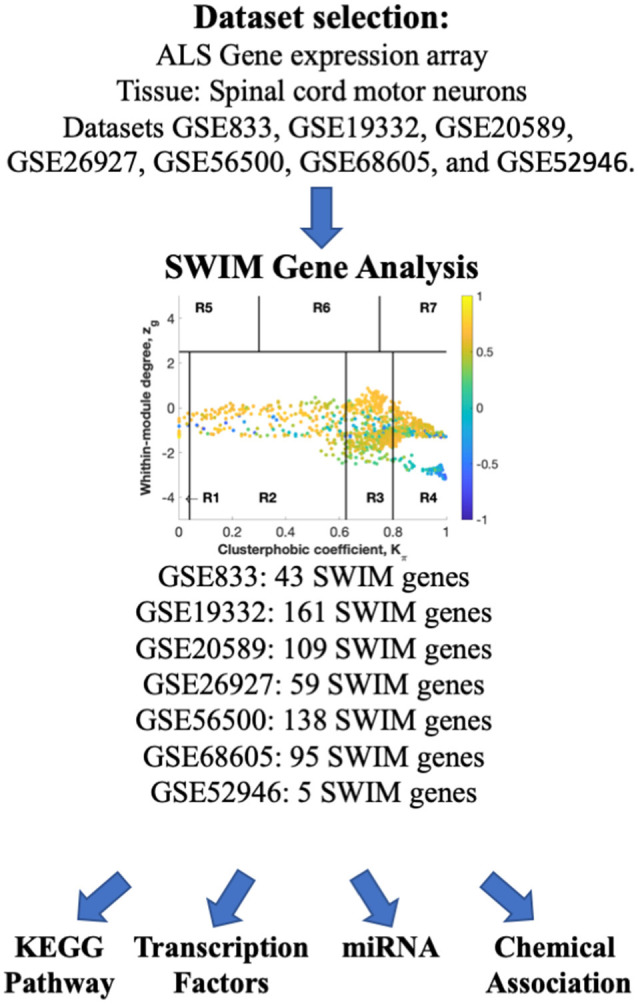
Overall study design. ArrayExpress and NCBI GEO databases were searched for human transcriptomic studies in ALS. SWIM analysis was performed to identify switch genes, which were further analyzed for functional pathways, regulatory transcription factors, miRNAs, and chemical associations using NetworkAnalyst.

We interrogated the Array Express and NCBI GEO databases to identify gene expression studies from ALS patients and age-matched controls. We focused our analysis on spinal cord motor neurons studies containing samples from ALS patients. As a result, seven gene expression data sets from ALS patients and age-matched healthy controls were identified and considered further (Tables s 1, 2). The overall strategy of the study is illustrated in Figure 1.

### Identification of Switch Genes in Spinal Cord Motor Neurons From ALS Patients

**Figure 2:**
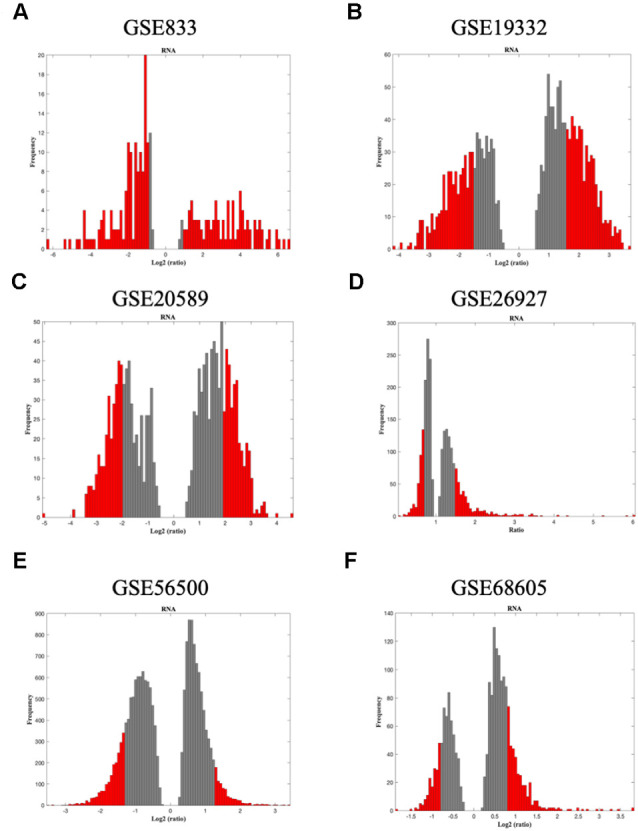
The panels **(A–F)** represent the results for GSE833, GSE19332, GSE20589, GSE26927, GSE56500, and GSE68605, respectively. Distribution of log2 fold change values where the red bars are selected for further analysis. The x-axis represents the fold-change value (log2 of the fold-change) that is the ratio of the average expression data in ALS patients compared to the average expression data in normal controls computed for protein-coding and non-coding RNAs. The y-axis represents the frequency of the obtained fold-change values. The gray bars represent the fold-change values associated with protein-coding and non-coding RNAs that will be discarded according to the selected threshold. The red bars represent the fold-change values associated with protein-coding and non-coding RNAs that were retained for further analysis.

**Figure 3:**
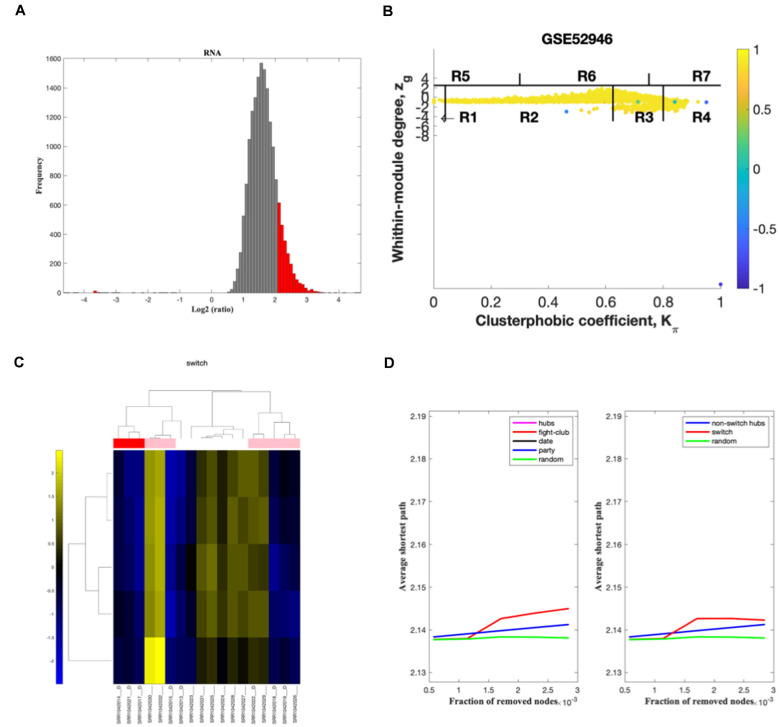
GSE52946 SWIM analysis. **(A)** Distribution of log2 fold change values where the red bars are selected for further analysis. **(B)** Heat cartography maps of nodes of the ALS/healthy correlation. **(C)** Dendrogram and heat map for switch genes. The suffix D indicates the samples from the ALS cohort. The colors represent expression levels, with blue indicating downregulated and yellow indicating upregulated. **(D)** Robustness of the correlation network.

The raw gene expression datasets from spinal cords motor neuron tissue from ALS patients were imported into SWIM. Then, the analysis was performed comparing ALS to healthy controls. In the first step, genes were retained (red bars) or eliminated (gray bars) according to the selected fold-change threshold (Figures 2, 3A).

**Figure 4:**
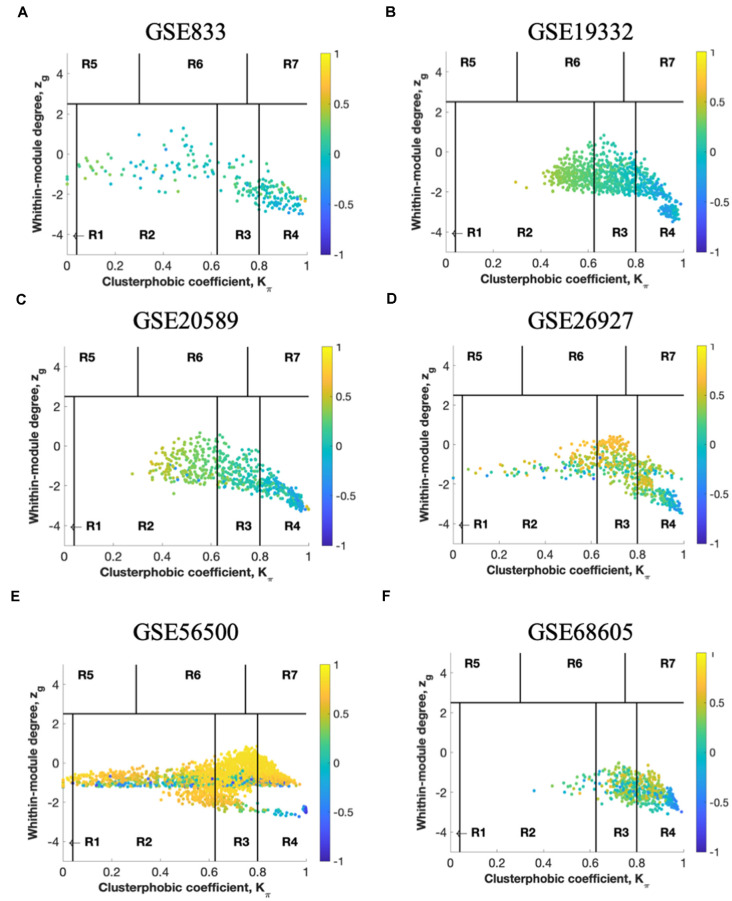
The panels **(A–F)** represent the results for GSE833, GSE19332, GSE20589, GSE26927, GSE56500, and GSE68605, respectively. Heat cartography map with nodes colored by their average Pearson Correlation Coefficient (APCC) value. Yellow nodes are party and date hubs, which are positively correlated in expression with their interaction partners. Blue nodes are the fight-club hubs, with an average negative correlation in expression with their interaction partners. Blue nodes falling in the region R4 are the switch genes characterized by low *Zg* and high *Kπ* values and are connected mainly outside their module. Thus, region R4 represents the switch genes.

In the second step, the average Pearson correlation coefficient allowed the identification of correlation communities (Figures 3B, 4). The nodes with a negative correlation value with their interaction partner, known as fight-club hubs, are depicted in R4 in blue. Two parameters identify the plane: *Zg* (within-module degree) and *Kπ* (clusterphobic coefficient), and it is divided into seven regions, each defining a specific node role (R1-R7). High *Zg* values correspond to nodes that are hubs within their module (local hubs), whereas low *Zg* values correspond to nodes with few connections within their module (non-hubs within their communities, but they could be hubs in the network). Each node is colored according to its average Pearson Correlation coefficient (APCC) value. Region R4 represents the switch genes.

**Figure 5:**
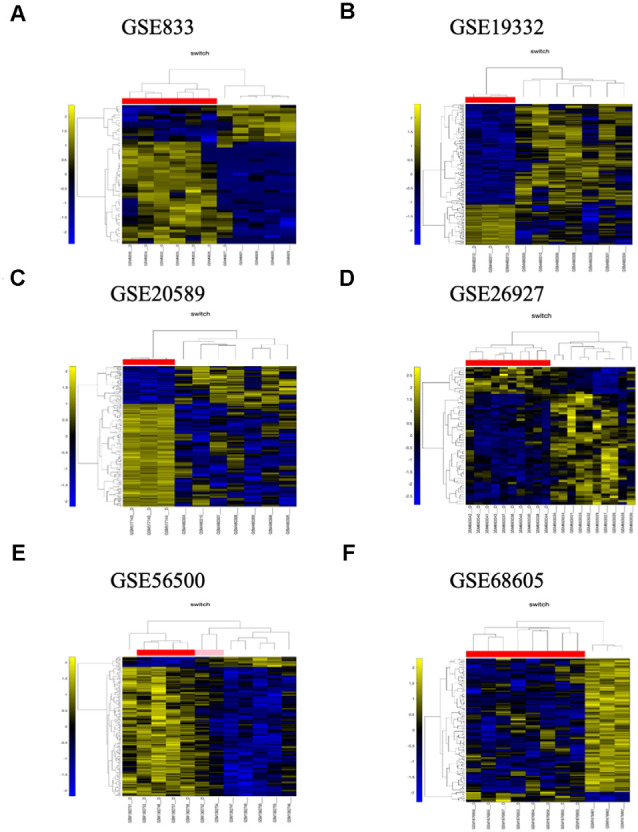
The panels **(A–F)** represent the results for GSE833, GSE19332, GSE20589, GSE26927, GSE56500, and GSE68605, respectively. Dendrogram and heat map analysis for switch genes. The colors represent different expression levels that increase from blue to yellow. The samples marked with a D after the number are the ones from the diseased cohort. The red, pink, and white bar at the top is an alternate marker of the cohorts. When the sample size is large the x-axis, labels are disabled. Red and pink denote ALS samples.

In the third step, the expression profiles of switch genes are clustered according to rows (switch genes) and columns (samples) of the switch genes expression data (biclustering; Figures 3C, 5). Switch genes identified from the arrays GSE833, GSE20589, and GSE56500 were primarily upregulated in ALS subjects, whereas the ones identified from the arrays GSE19332 and GSE26927 mainly were downregulated.

**Figure 6:**
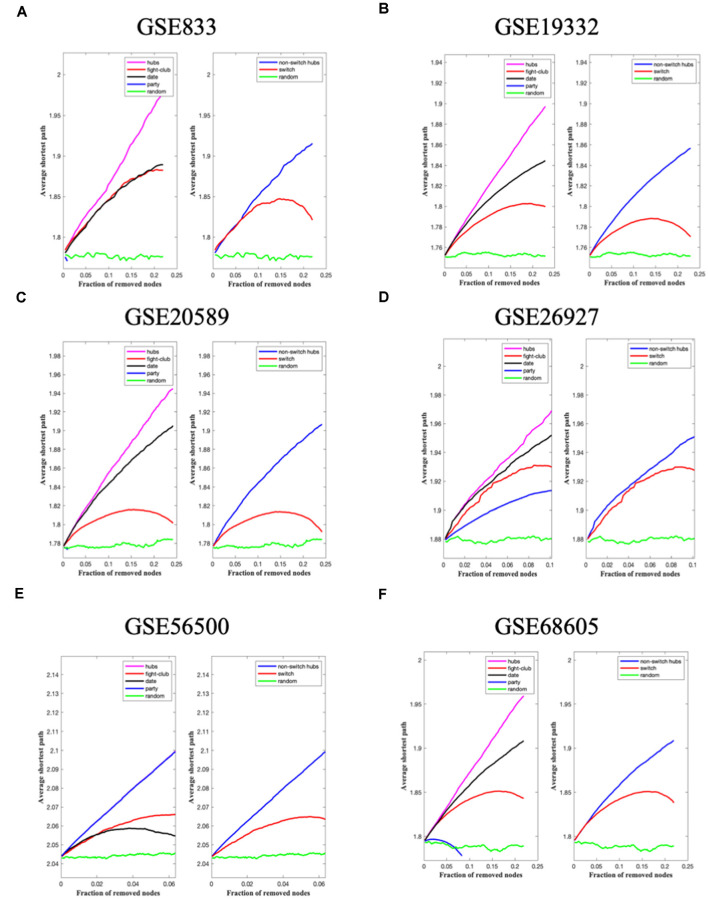
The panels **(A–F)** represent the results for GSE833, GSE19332, GSE20589, GSE26927, GSE56500, and GSE68605, respectively. Robustness of the correlation network. The x-axis represents the cumulative fraction of removed nodes, while the y-axis represents the average shortest path. The shortest path between two nodes is the minimum number of consecutive edges connecting them. Thus, each curve corresponds to the variation of the average shortest path of the correlation network as a function of the removal of nodes specified by the colors of each curve.

In the final step, the robustness of the analysis is determined (Figures 3D, 6). Fight-club hubs differ from date and party hubs, and switch genes are significantly different from random, confirming the analysis’s robustness.

SWIM analysis identified 14, 161, 109, 59, 137, 95, and 5 switch genes for the datasets GSE833, GSE19332, GSE20589, GSE26927, GSE56500, GSE68605, and GSE52946 respectively. The switch genes identified in the seven datasets are listed in Supplementary Table 1. A Venn diagram analysis performed on the switch genes identified from the seven datasets showed limited overlapping. No switch genes were shared between three or more datasets. Ten genes were shared between two datasets: ASPM, DNTTIP1, ENC1, PABPC1L, POR, and ZNF688, AVL9, FABP6, HOXDA, and ZNF649.

### Pathway Enrichment Analysis

Switch genes obtained from the seven datasets were imported into NetworkAnalyst to identify the associated functional role and biological processes. This analysis identified 51, 36, 53, 48, 86, 43, and 10 pathways associated with the switch genes from the datasets GSE833, GSE19332, GSE20589, GSE26927, GSE56500, GSE68605, and GSE52946, respectively (Supplementary Table 2). The top three pathways obtained with each set of switch genes are listed in Table 3. A Venn diagram analysis was performed to determine the overlapping pathways. Interestingly, six pathways were shared between six datasets: viral carcinogenesis, PI3K-Akt signaling pathway, focal adhesion, proteoglycans in cancer, colorectal cancer, and thyroid hormone signaling. There were no shared pathways with the dataset GSE52946. Switch genes identified in the dataset GSE52946 were enriched in several pathways including, proteasome, legionellosis, PPAR signaling, antigen processing, and presentation, peroxisome, IL-17 signaling, prostate cancer, progesterone-mediated oocyte maturation, Th17 cell differentiation, and oxidative phosphorylation.

**Table 3:** Top three pathways identified from each ALS/healthy switch genes analysis.

Pathways	*P*.Value	FDR
**GSE833**		
Pathways in cancer	2.96E-11	9.43E-09
Viral carcinogenesis	8.83E-10	1.40E-07
Proteoglycans in cancer	3.48E-08	3.69E-06
**GSE19332**		
Endometrial cancer	5.94E-04	6.89E-02
Gap junction	5.94E-04	6.89E-02
Cell cycle	6.5E-04	6.89E-02
**GSE20589**		
Neurotrophin signaling	8.36E-05	2.04E-02
HTLV-I infection	1.28E-04	2.04E-02
Viral carcinogenesis	3.88E-04	2.65E-02
**GSE26927**		
Prostate cancer	1.22E-05	3.87E-03
Adherens junction	7.52E-05	1.2E-02
Jak-STAT signaling	1.43E-04	1.39E-02
**GSE56500**		
Pathways in cancer	2.96E-11	9.43E-09
Viral carcinogenesis	8.83E-10	1.40E-07
Proteoglycans in cancer	3.48E-08	3.69E-06
**GSE68605**		
PI3K-Akt signaling pathway	5.88E-05	1.63E-02
Glioma	1.02E-04	1.63E-02
Proteoglycans in cancer	2.45E-04	2.56E-02
**GSE52946**		
Proteasome	3.44E-02	1
Legionellosis	4.19E-02	1
PPAR signaling pathway	5.60E-02	1

### Gene-Transcription Factor Interaction Analysis

To identify the master regulators of switch genes, transcription factor analysis was performed on NetworkAnalyst using three different databases (ENCODE, ChEA, and JASPAR). A Venn diagram analysis identified the transcription factors shared between the three databases interrogated (Supplementary Table 3). This analysis yielded 4, 20, 21, 11, 18, and 18 transcription factors associated with the switch genes from the datasets GSE833, GSE19332, GSE20589, GSE26927, GSE56500, and GSE68605, respectively. No transcription factors were shared between the three databases used to analyze the switch genes obtained from the array GSE52946. In addition, Venn diagram analysis identified EGR1, ELK1, PPARG, GATA2, CREB1, STAT3, and CEBPB as shared transcription factors regulating the switch genes obtained from six different arrays (Table 4). Furthermore, SREBF2, RELA, and YY1 were shared between five datasets.

**Table 4:** Transcription factors shared between at least three arrays.

	**Arrays**	**Transcription factors**
Shared between 6 arrays
	GSE833, GSE19332, GSE20589, GSE56500, GSE68605, and GSE26927	EGR1, ELK1, PPARG, GATA2, CREB1, STAT3, CEBPB
Shared between 5 arrays
	GSE19332, GSE20589, GSE26927, GSE56500, and GSE68605	SREBF2
	GSE833, GSE19332, GSE20589, GSE56500, and GSE68605	RELA, YY1
Shared between 4 arrays
	GSE19332, GSE26927, GSE56500, and GSE68605	STAT1
	GSE19332, GSE20589, GSE56500, and GSE68605	GATA3
Shared between 3 arrays
	GSE833, GSE20589, and GSE68605	ARNT, MYC
	GSE833, GSE20589, and GSE56500	JUN
	GSE833, GSE19332, and GSE56500	E2F4
	GSE833, GSE19332, and GSE20589	SREBF1
	GSE19332, GSE20589, and GSE26927	GATA1

### Gene-miRNA Interaction Analysis

To further study the regulation of the switch genes, a gene-miRNA interaction network analysis was performed in NetworkAnalyst using two different databases (TarBase v.8.0. and miRtarBase v.8.0.). We identified 4, 66, 45, 11, 73, 36, and 1 miRNAs associated with the switch genes from the datasets GSE833, GSE19332, GSE20589, GSE26927, GSE56500, GSE68605, and GSE52946 respectively (Supplementary Table 4). Venn diagram analysis identified mir-335-5p, mir-16-5p, mir-218-5p, mir-124-3p, let-7a-5p, and mir-155-5p as key shared miRNAs regulating the switch genes obtained from the six different arrays (Table 5). In addition, 11 miRNAs were involved in the regulation of the switch genes in five different datasets.

**Table 5:** miRNAs shared between at least three arrays.

	**Arrays**	**miRNAs**
Shared between 6 arrays
	GSE833, GSE19332, GSE20589, GSE26927, GSE56500, and GSE68605	mir-335-5p, hsa-mir-16-5p, hsa-mir-218-5p, hsa-mir-124-3p, and hsa-let-7a-5p
	GSE19332, GSE20589, GSE26927, GSE56500, GSE68605, and GSE52946	hsa-mir-155-5p
Shared between 5 arrays
	GSE19332, GSE20589, GSE26927, GSE56500, and GSE68605	hsa-mir-24-3p and hsa-mir-7-5p
	GSE833, GSE19332, GSE20589, GSE56500, and GSE68605	let-7b-5p, hsa-mir-93-5p, hsa-mir-1-3p, hsa-mir-15a-5p, hsa-mir-192-5p, hsa-mir-103a-3p, hsa-mir-27a-3p, hsa-mir-26b-5p
	GSE833, GSE19332, GSE20589, GSE26927, and GSE56500	hsa-mir-128-3p
Shared between 4 arrays
	GSE19332, GSE20589, GSE56500, and GSE68605	hsa-mir-30a-5p, hsa-mir-17-5p, hsa-mir-122-5p, hsa-mir-20a-5p, hsa-mir-26a-5p, hsa-mir-21-5p, hsa-mir-34a-5p, and hsa-mir-203a-3p
Shared between 3 arrays
	GSE20589, GSE56500, and GSE68605	hsa-mir-23b-3p and hsa-mir-375
	GSE833, GSE26927, and GSE68605	hsa-mir-29a-3p
	GSE833, GSE56500, and GSE68605	hsa-mir-142-3p
	GSE19332, GSE20589, and GSE56500	hsa-mir-92a-3p, hsa-mir-484, hsa-mir-744-5p, hsa-mir-19a-3p, and hsa-mir-31-5p
	GSE19332, GSE56500, and GSE68605	hsa-mir-186-5p, hsa-mir-181a-5p, and hsa-mir-101-3p

To investigate the functional role of miRNAs in ALS, we performed a pathway analysis using miTALOS 2.0, a web-based platform for miRNAs functional analysis (Kowarsch et al., 2011; Preusse et al., 2016). Functional analysis of the miRNAs regulating the switch genes was associated with insulin signaling, signaling pathways regulating pluripotency of stem cells, and renal cell carcinoma (Supplementary Table 5).

### Protein-Chemical Interaction Analysis

We next investigated chemicals interacting with the switch genes. To this end, we performed a protein-chemical network analysis importing the switch genes into NetworkAnalyst. Each dataset of switch genes was analyzed separately. This network analysis resulted in 22, 129, 109, 50, 118, and 83 chemicals associated with the switch genes from the datasets GSE833, GSE19332, GSE20589, GSE26927, GSE56500, and GSE68605, respectively (Supplementary Table 6). No chemical was associated with the switch genes obtained from the dataset GSE52946. Interestingly, 25 chemicals were shared between the six analyses (Supplementary Table 6). Valproic acid, cyclosporine, aflatoxin B1, benzo(a) pyrene, 4-(5-benzo(1,3) dioxol-5-yl-4-pyridin-2-yl-1H-imidazol-2-yl)benzamide, silicon dioxide, estradiol, acetaminophen, copper sulfate, arsenic, nickel, benzo(a)pyrene and quercetin were among the shared chemicals. Chemicals were ranked according to network topology measurements, including degree and betweenness centrality.

## Discussion

### Identification of Switch Genes and Dysregulated Pathways in Spinal Cord Motor Neurons of ALS Patients

In this study, we built co-expression networks using SWIM software to identify genes associated with the transition from healthy status to ALS. SWIM analysis identified a total of 750 switch genes in six independent microarrays containing transcriptomic data from spinal motor neurons in ALS patients. Venn diagram analysis yielded a limited overlap of switch genes between the different arrays. These results may be explained by the differences in the microarray platforms and methods as well as genetic differences between ALS patients. For example, GSE19332, GSE20589, and GSE68605 used motor neurons from ALS subjects with CHMP2B, SOD1, and C9ORF72 mutations, respectively. In addition, GSE833, GSE56500, and GSE52946 datasets used a combination of sporadic and familial ALS cases.

Biological and functional analysis of switch genes identified six pathways, including viral carcinogenesis, PI3K-Akt signaling pathway, focal adhesion, proteoglycans in cancer, colorectal cancer, and thyroid hormone signaling, shared between the six datasets. A potential link between viral carcinogenesis and motor neuron degeneration is intriguing, but unfortunately, few studies have investigated this association. The human endogenous retrovirus (HERV)-K was reported to interfere with RNA-binding and alternative splicing regulation, processes known to play a role in neurodegeneration. For instance, CRISPR/Cas9 gene-editing of HERV-K reduced the expression of SF2/ASF (splicing factor 2/alternative splicing factor) and TDP-43 mRNA levels *in vitro* (Ibba et al., 2018). Recently, switch genes identified in the blood of ALS were enriched in pathways related to viral infection (Santiago et al., 2021). Interestingly, switch genes identified in the female datasets were associated with the Epstein-Barr virus, hepatitis B, and hepatitis C. The link between viral infection and carcinogenesis and ALS warrants further investigation.

In addition to viral infection and carcinogenesis, the PI3K-AKT pathway was identified as one of the most significant pathways. Consistent with these findings, several studies have identified the PI3K-AKT pathway as a critical mechanism in the pathogenesis of ALS. For example, a network analysis of genes dysregulated in the motor cortex and spinal motor neurons in ALS identified the PI3K-AKT as the most significant pathway (Recabarren-Leiva and Alarcon, 2018). Another study reported that dysregulated expression of astrocyte elevated gene 1 (AEG1) and the inhibition of the PI3K-AKT pathway were associated with cell death in ALS motor neurons (Yin et al., 2015). Moreover, PI3K-AKT was the most significantly enriched pathway identified in switch genes obtained from males with ALS (Santiago et al., 2021). These studies suggest that targeting the PI3K-AKT pathway may be a possible therapeutic route in ALS. Also, studies investigating sex-specific differences in the involvement of these pathways may provide new insights into personalized therapies for ALS patients.

Thyroid function has been investigated in several studies of ALS. For example, higher levels of thyroxine correlated with prolonged survival in the SOD1-G93A mouse model of ALS (Li et al., 2012) . Contrary to these findings, thyroid hormone levels did not correlate with prolonged survival in a clinical study including 278 patients with ALS (Zheng et al., 2014). Another study reported the expression of protein μ crystalline (CRYM), a key regulator of thyroid hormone transport, is reduced in the corticospinal tract of ALS patients (Hommyo et al., 2018). Based on these studies, there is no conclusive evidence between thyroid dysfunction and the development of ALS. Future molecular and clinical studies are needed to investigate further the association between thyroid hormone signaling and ALS.

Analysis of the high throughput RNA sequencing study GSE52946 yielded five switch genes: MTRNR2L1, MTRNR2L10, MTRNR2L2, MTRNR2L3, and MTRNR2L8. Functional analysis revealed these genes associated with the proteasome, legionellosis, PPAR signaling, antigen processing and presentation, peroxisome, IL-17 signaling, prostate cancer, progesterone-mediated oocyte maturation, Th17 cell differentiation, and oxidative phosphorylation. Some of these switch genes have been implicated in processes related to ALS. For example, MTRNR2L1 is associated with inflammation, oxidative stress, and insulin resistance (Veilleux et al., 2015). In this context, several epidemiological studies have suggested a link between diabetes and ALS. Interestingly, type 2 diabetes has been found to be protective against ALS in several populations but the molecular mechanisms underlying this neuroprotection remain unclear (Kioumourtzoglou et al., 2015; D’Ovidio et al., 2018; Wannarong and Ungprasert, 2020; Ferri et al., 2021). Another switch gene, MTRNR2L8, has been implicated in atherosclerotic stroke and it may be a therapeutic target and a diagnostic biomarker for stroke (Shen et al., 2019; Wong et al., 2021). In this regard, cardiovascular disease and atherosclerosis is associated with a higher risk of ALS in some populations (Kioumourtzoglou et al., 2016; Garton et al., 2021). Similarly, RNR1 is associated with dilated cardiomyopathy (Schiano et al., 2021). Future prospective longitudinal studies will be important to determine the validity of the association between diabetes, cardiovascular disease, and ALS.

### Switch Genes Expression Regulation

Network analysis identified 18 transcription factors regulating the switch genes from the six datasets: GATA2, ELK1, EGR1, PPARG, CREB1, SREBF2, CEBPB, STAT1, STAT3, YY1, RELA, GATA3, ARNT, GATA1, KLF4, SREBF1, JUN, and E2F4. Among these transcription factors, ELK1 and GATA2 were shared between six out of seven arrays. Although there is no direct evidence linking GATA2 and ELK1 with ALS, these transcription factors have been associated with dementia and neurodegeneration. For instance, ELK1 has been implicated in early epigenetic changes and neuroprotection in cellular models of Huntington’s disease (Anglada-Huguet et al., 2012; Yildirim et al., 2019). Interestingly, a neurotoxic form of ELK1 is associated with the development of neuronal inclusions in several neurodegenerative disorders including Lewy body, Alzheimer’s, and Huntington’s diseases (Sharma et al., 2010). In addition, network analysis identified GATA2 as an important transcription factor regulating transcriptomic changes in Alzheimer’s disease (Rahman et al., 2019, 2020).

Several of the transcription factors identified have been implicated in the pathogenesis of ALS. For example, EGR1, a transcription factor known to play a role in cellular division, neuronal plasticity, and memory, has been proposed to be associated with ALS risk (Recabarren-Leiva and Alarcon, 2018). The involvement of PPARG in neuroprotection has been extensively documented in neurodegenerative diseases (Kiaei, 2008; Prashantha Kumar et al., 2020). Activation of PPARG has been shown to elicit neuroprotection in drosophila models overexpressing TDP-43 or FUS and in SOD mice models (Kiaei et al., 2005; Joardar et al., 2015; Rodriguez-Cueto et al., 2018). The neuroprotective effects of PPARG may be mediated through its actions against lipid peroxidation (Benedusi et al., 2012). Despite this evidence, a randomized trial using pioglitazone, a PPARG agonist, in combination with riluzole, showed no beneficial effects on the survival of ALS patients (Dupuis et al., 2012). In addition, E2F4 is another transcription factor associated with ALS. For example, differentially expressed genes in induced pluripotent stem cells derived from C9ORF72-ALS patients were regulated by E2F4/DREAM complex (Wong and Venkatachalam, 2019).

Interestingly, CREB and SREBF2 have been documented to interact with TDP3, a major constituent of neuronal inclusions in ALS and frontotemporal dementia. Dysfunctional TDP-43 reduced dendritic branching of cortical neurons through inhibition of CREB transcriptional activity (Yamashita et al., 2014; Herzog et al., 2020). Interaction of TDP43 with SREBF2, an important transcription factor involved in cholesterol metabolism, mediated oligodendrocyte myelination, and cholesterol homeostasis (Ho et al., 2021). Bioinformatic analysis of gene expression datasets of motor neurons from sporadic ALS patients identified RelA and NF-κB1 as key hub genes involved in regulating the extracellular matrix structure and function (Lin et al., 2020). Persistent activation and nuclear translocation of STAT3 were observed in the spinal cord of ALS patients and a SOD1-mice model of ALS (Shibata et al., 2009, 2010; Ohgomori et al., 2017). It has been proposed that activation of STAT3 could be a consequence of neuroinflammation, and its modulation could enhance motor neuron differentiation and thus be beneficial in neurotrauma and neurodegenerative diseases (Natarajan et al., 2014; Ohgomori et al., 2017).

We also investigated the regulation of switch genes by miRNAs. Six miRNAs, mir-335-5p, mir-16-5p, mir-218-5p, mir-124-3p, let-7a-5p, and mir-155-5p, were identified as shared regulators of the switch genes between six out of seven datasets. In addition, 11 miRNAs were shared between five datasets (mir-24-3p, mir-7-5p, let-7b-5p, mir-93-5p, mir-1-3p, mir-15a-5p, mir-192-5p, mir-103a-3p, mir-27a-3p, mir-26b-5p, and mir-128-3p). Several of these miRNAs are dysregulated in animal models and patients with ALS. For example, mir-16-5p, let-7a-5p, and mir-26-5p were significantly downregulated in serum samples from sporadic ALS patients suggesting their potential as diagnostic biomarkers (Liguori et al., 2018; Joilin et al., 2020). Interestingly, several animal studies have proposed a neuroprotective role of mir-16 in Alzheimer’s and prion diseases (Liu et al., 2012; Majer et al., 2012). Elevated levels of miR-124-3p in CSF exosomes correlated with disease severity in ALS patients (Yelick et al., 2020). Collectively, these results suggest that these miRNAs may be useful biomarkers; however, replication and further validation in several independent studies will be crucial to determine their diagnostic potential for ALS patients.

### Identification of Potential Therapeutics and Environmental Risk Factors for ALS

A protein-chemical interaction analysis identified 25 shared chemicals interacting with the switch genes in six datasets. Some of these chemicals may be useful therapeutic agents, whereas others may be detrimental and pose a potential risk for ALS. Valproic acid was the most highly ranked chemical in six datasets. Other shared chemicals were cyclosporine, aflatoxin B1, estradiol, acetaminophen, carbamazepine, copper sulfate, arsenic, nickel, quercetin, and benzo(a)pyrene. Several of these drugs have been investigated for the treatment of neurodegenerative disorders, including ALS. For example, valproic acid protected spinal cord motor neurons against glutamate toxicity and significantly prolonged the disease duration in an ALS mouse model (Sugai et al., 2004). In addition, the combination of valproic acid with lithium effectively delayed disease onset, attenuated neurological symptoms, and extended the lifespan in a mouse ALS model (Feng et al., 2008). The neuroprotective effects of valproic acid and lithium may be mediated *via* the activation of the Notch signaling pathway and the suppression of endoplasmic reticulum stress (Naganska et al., 2015; Wang et al., 2015a, b; Jiang et al., 2016). Nevertheless, other animal studies using valproic acid reported neuroprotective effects but not improvements in disease survival (Rouaux et al., 2007; Crochemore et al., 2009).

The evidence from clinical studies using valproic acid is also inconsistent. In a randomized trial, administration of valproic acid at similar doses used in epilepsy had no effects on survival or disease progression in patients with ALS (Piepers et al., 2009). Another study showed that the combination of valproic acid with lithium exerted neuroprotection and increased survival in ALS patients; however, the trial stopped due to the late adverse effects of treatment (Boll et al., 2014). Collectively, the mixed findings using valproic acid call for a larger randomized clinical trial to determine the efficacy in ALS patients.

Other potential drugs identified were cyclosporine, carbamazepine, acetaminophen, and estradiol. Cyclosporine A is a well-known immunosuppressive agent that has been shown to protect from mitochondrial neuronal death in traumatic brain injury and acute ischemic stroke (Gajavelli et al., 2015; Nighoghossian et al., 2016; Matsumoto et al., 2018). In the context of ALS, systemic administration of cyclosporine extends the lifespan of ALS transgenic mice (Keep et al., 2001; Kirkinezos et al., 2004). Carbamazepine is commonly used to control seizures in epilepsy and peripheral neuropathy caused by diabetes (Maan et al., 2021). Administration of carbamazepine delayed the disease onset and extended the lifespan in the SOD1-G93A mouse ALS model (Zhang et al., 2018). To the best of our knowledge, neither cyclosporine nor carbamazepine has been tested in clinical trials for ALS.

Similar to our results, a network analysis of differentially expressed genes in ALS patients’ motor cortex and spinal cord identified acetaminophen, estradiol, progesterone, and resveratrol as possible therapeutics (Recabarren and Alarcon, 2017; Recabarren-Leiva and Alarcon, 2018). The clinical evidence about the therapeutic effect of acetaminophen in ALS patients is not clear. For example, a recent meta-analysis showed a reduced occurrence of ALS in patients taking acetaminophen but not aspirin (Chang et al., 2020). Nonetheless, the authors noted that the analyzed studies did not control for past medical history and drug dosages, which may have confounded their results. Contrary to these findings, another study concluded that intake of aspirin or NSAIDs might shorten disease survival in ALS patients (Qureshi et al., 2008). Further clinical studies are needed to better understand the effects of these drugs on ALS patients.

Interestingly, estradiol was among the shared drugs identified in the protein-drug interaction analysis. In this regard, sex-specific differences have been linked to the pathogenesis of ALS. Epidemiological studies from different populations indicate that men are at higher risk of ALS than women (McCombe and Henderson, 2010; Couratier et al., 2016). One earlier study showed that men are at a higher risk of ALS at a younger age but there is an equal risk between men and women above 60 years old (Haverkamp et al., 1995). In this context, it has been proposed that estrogens may exert neuroprotection in the brain and spinal motor neurons (Nakamizo et al., 2000; Johann et al., 2011; Cardona-Rossinyol et al., 2013). The neuroprotective effects of estrogen may be mediated through gene expression regulation and its antioxidant and mito-protectant activities (Behl et al., 2000; Dykens et al., 2005). Several studies showed that ovariectomy accelerated disease progression, and treatment with a high dose of 17beta-estradiol reversed these effects in SOD1 transgenic mice (Groeneveld et al., 2004; Choi et al., 2008). Similarly, raloxifene, a non-steroidal benzothiophene used in the treatment of osteoporosis, mediates neuroprotection through estrogen receptors (Arevalo et al., 2011). The neuroprotective effect of raloxifene was confirmed in a cellular model of ALS (Zhou et al., 2018). Consistently with these findings, our previous study identified sex-specific switch genes and pathways in the blood of ALS patients. Notably, estradiol was found among the potential drugs for females with ALS (Santiago et al., 2021). The possible neuroprotective effects of estradiol warrant further investigation in larger clinical trials. These studies reinforce the importance of understanding sex differences in the development of personalized treatments for ALS patients.

Furthermore, other chemicals interacting with the switch genes were quercetin, copper sulfate, arsenic, nickel, and benzo(a)pyrene. Notably, some of these chemicals may be neuroprotective, whereas others may increase the risk for ALS. For example, quercetin, a naturally occurring flavonoid, has been shown to be effective against SOD1 fibrillation in biochemical experiments and animal models of ALS (Ip et al., 2017; Bhatia et al., 2020). In addition, quercetin attenuated aluminum-induced neurodegeneration in the rat hippocampus (Sharma et al., 2016).

Unlike the neuroprotective effects of quercetin, exposure to heavy metals and organic compounds like copper, arsenic, nickel, and benzo(a)pyrene is associated with neurodegeneration. Elevated levels of metals including aluminum, copper, lead, and arsenic have been found in the blood and CSF of ALS patients (Callaghan et al., 2011; Peters et al., 2016; Patti et al., 2020). Similarly, exposure to benzo(a)pyrene* in utero* has been associated with alpha synuclein toxicity and microglial activation in mice (Xu et al., 2021). Further, benzo(a)pyrene exposure is associated with cognitive decline and exacerbation of Alzheimer’s disease pathology in mice (Liu et al., 2020). Exposure to electronic waste containing nickel and other metals is associated with an increased risk of neurodegeneration (Zhu et al., 2021). Environmental exposure to heavy metals is associated with neurotoxic effects on astrocytes, key mediators of cellular and metabolic homeostasis in the central nervous system (Li et al., 2021). Collectively, the analysis of chemical-switch genes interaction provides information about potential therapeutics, neuroprotective agents, and hazardous exposure to chemicals that may trigger neurodegeneration.

## Limitations

The results from this study should be taken with caution for several reasons. Firstly, the findings presented in this study are the result of bioinformatic analyses using different databases and software that relies on the accuracy of publicly available deposited microarrays. Differences in array platforms, curation methods, and pre-processing of arrays may have introduced bias. In regards to the sample characteristics, some of the arrays contained data from ALS patients with different mutations and sporadic cases and thus differences in genetic factors could have impacted the results. These differences in array platforms and methods may have accounted for the non-overlap of switch genes with the RNA sequencing dataset GSE52946. In addition, motor neurons were collected from different locations in the spinal cord, which may have introduced bias. Another limitation is the use of post-mortem tissues, which may imply the use of ALS subjects in the late stages of the disease. The switch genes and pathways identified may be a representation of the molecular disturbances occurring in advanced stages of ALS. The use of tissues from pre-symptomatic and early stages ALS subjects would be ideal to capture the dysregulated molecular pathways in the early stages of the disease. SWIM analysis by sex would have been ideal to determine sex-specific differences in pathways and switch genes regulation but unfortunately, the arrays did not contain enough samples from both males and females. Future mechanistic studies using cellular and animal models to investigate the functional role of the switch genes and the therapeutic benefit of the drugs identified will be essential to confirm the results presented in this study.

## Conclusions

Analysis of co-expression networks implemented by SWItch Miner software could be useful in identifying the molecular pathways disrupted in ALS. One advantage of this method is the identification of switch genes whose expression is associated with drastic transcriptional changes that may lead to ALS. In this study, we identified switch genes in the spinal motor neurons of ALS patients. Switch genes were enriched in viral carcinogenesis, cell cycle, PI3K-Akt, focal adhesion, proteoglycans in cancer, colorectal cancer, and thyroid hormone signaling. Specifically, pathways related to viral carcinogenesis and hepatitis have been identified in transcriptomic studies from the blood of ALS patients (Santiago et al., 2021). The precise linkage between viral infection and the development of ALS has not been thoroughly investigated. This previously unrecognized association between viral infection and ALS could lead to the discovery of potential new treatments. Future studies will be aimed at identifying shared pathways and master regulators between virally transmitted diseases and ALS. The chemical analysis identified several potential therapeutics, neuroprotective agents, and potentially hazardous metals and chemical compounds associated with neurodegeneration.

## Data Availability Statement

The original contributions presented in the study are included in the article/Supplementary Material, further inquiries can be directed to the corresponding author.

## Author Contributions

VB, JS, JQ, and JP conceived and contributed to the designs of the methods used in this study, analyzed the data, and wrote the article. All authors contributed to the article and approved the submitted version.

## Funding

This study was funded by the National Institute on Aging (NIA) grant number R01AG062176 to JP. In addition, funds were provided by Rosalind Franklin University of Medicine and Science.

## Conflict of Interest

JS is the founder of and employed by NeuroHub Analytics, LLC. JQ is the founder of and employed by Q Regulating Systems, LLC. The remaining authors declare that the research was conducted in the absence of any commercial or financial relationships that could be construed as a potential conflict of interest.

## Publisher’s Note

All claims expressed in this article are solely those of the authors and do not necessarily represent those of their affiliated organizations, or those of the publisher, the editors and the reviewers. Any product that may be evaluated in this article, or claim that may be made by its manufacturer, is not guaranteed or endorsed by the publisher.

## Footnotes

^1^ https://www.ncbi.nlm.nih.gov/gds

^2^ https://www.ebi.ac.uk/arrayexpress/

## Supplementary Materials

Supplementary Table 1 Switch genes.

Supplementary Table 2 Pathways analysis.

Supplementary Table 3 Transcription factors-Gene interactions analysis.

Supplementary Table 4 Gene-miRNA interactions analysis.

Supplementary Table 5 miRNA pathways.

Supplementary Table 6 Protein-chemicals interactions analysis.
